# Developing an integrated HIV clinical and population database in rural south Africa

**DOI:** 10.23889/ijpds.v9i5.2830

**Published:** 2024-09-18

**Authors:** Dickman Gareta, Evelyn Lauren, Khumbo Shumba, Kobus Herbst, Dorina Onoya, Jacob Bor

**Affiliations:** 1 Africa Health Research Institute; 2 Department of Biostatistics, Boston University School of Public Health; 3 Health Economics and Epidemiology Research Office, Department of Internal Medicine, School of Clinical Medicine, Faculty of Health Sciences, University of the Witwatersrand; 4 DSI-SAMRC South African Population Research Infrastructure Network; 5 Department of Epidemiology, Boston University School of Public Health; 6 Department of Global Health, Boston University School of Public Health, Boston

## Background

HIV cohort data are crucial for understanding the effects of HIV infection and treatment. Linkage between HIV clinical and population records unlocks potential to understand the cause and consequences of social-economic factors on HIV-care seeking behaviors and mortality outcomes. We created HIV data infrastructure by linking HIV treatment records with routinely collected records.

## Methods

We linked HIV cohort data(n=119,434) to Health and Demographic Surveillance(n=256,425), laboratory tests(n=617,314), clinic visits and hospital admissions(n=170,325) from KwaZulu-Natal, an HIV-endemic setting in rural South Africa. We used graph-based probabilistic record linkage algorithm to link the data for HIV patients accessing care at 17 clinics within the Hlabisa sub-district. Africa Health Research Institute (AHRI) has collected health and demographic surveillance data since 2000. AHRI has set up a hospital information system in the district to capture ICD-10 code admissions and discharges.

## Results

From the 5% sample of 462,524 linkage clusters, the computed F-score was 0.88, sensitivity was 88% and positive predictive value was 91%. 38.5% of the patient ID clusters contained records linked across multiple databases. 36% of the HIV patient ID clusters contained records linked with laboratory tests. 19.5% of the HIV patient ID clusters contained data on demographic surveillance. 12.5% of the HIV patient ID clusters contained records linked with clinic visits and hospital admissions. 4% of patient IDs contained records across all four databases.

## Conclusion

We have successfully created a cross-sectorial HIV cohort through probabilistic record linkage methods. The cohort provides a platform to answer policy-relevant questions.

